# High Potassium Application Rate Increased Grain Yield of Shading-Stressed Winter Wheat by Improving Photosynthesis and Photosynthate Translocation

**DOI:** 10.3389/fpls.2020.00134

**Published:** 2020-02-28

**Authors:** Yi Wang, Zhongkui Zhang, Yuanyuan Liang, Yulong Han, Yanlai Han, Jinfang Tan

**Affiliations:** ^1^ College of Resources and Environment, Henan Agricultural University, Zhengzhou, China; ^2^ State Key Laboratory of Wheat and Maize Crop Science, Henan Agricultural University, Zhengzhou, China; ^3^ Collaborative Innovation Center of Henan Grain Crops, Henan Agricultural University, Zhengzhou, China

**Keywords:** chlorophyll fluorescence characteristics, dry matter translocation amount, grain yield, potassium application rate, shading stress

## Abstract

Wheat (*Triticum aestivum* L) production on the Huang-Huai Plain of China has substantially affected in the past 50 years as a result of the decreasing total solar radiation and sunshine hours. Potassium has a significant effect on improving leaf photosynthesis ability under stress conditions. Five potassium application rates (K), 0 (K0), 50 (K50), 100 (K100), 150 (K150), and 250 (K250) mg K_2_O kg^-1^ soil, combined with two shading levels, no shading (NS) and shading at early filling stage for 10 days (SE), were used to investigate the effects of K application on winter wheat growth under SE condition. Under NS condition, the parameters related to chlorophyll fluorescence characteristics, dry matter productivity and grain yields reached the maximum values at a middle K application rate (100 mg K_2_O kg^-1^ soil). Shading stress significantly reduced leaf SPAD value, showed negative effects on chlorophyll fluorescence characteristics and reduced grain yield of winter wheat. However, as the result of the interaction of K×S, compared to NS condition, higher K application rate (150 mg and 250 K_2_O kg^-1^ soil) was beneficial in terms of achieving a higher grain yield of winter wheat under SE by improving leaf SPAD value, alleviating the damage of SE on the winter wheat photosynthetic system, and increasing fructan content and dry matter translocation percentage.

## Introduction

According to available data, wheat yield has been substantially affected in the Huang-Huai Plain as a result of the decreasing total solar radiation and sunshine hours in the middle and lower regions of the Yellow River basin in the past 50 years (Yu et al., 2002; Yang et al., 2005; Duan et al., 2018). Moreover, this reduction is more pronounced in winter and summer seasons (Xu and Zhao, 2005; Yang et al., 2005), especially after the wheat flowering, due to recurrent rainy climate that results in lower light intensity (Wang et al., 2003; Liu et al., 2014). Since serious potassium (K) deficiency still often exists in light loamy or sandy soil, potash fertilizer application was often needed in order to improve yield and quality of wheat in loam soil type (Andersson et al., 2007; Wang et al., 2008; Pettigrew, 2008; Grzebisz et al., 2013).

Previously, much research has been conducted on the effects of low light intensity stress or K fertilizer application on crop photosynthetic characteristics and crop dry matter production characteristics. The results demonstrated that shading reduced the nitrogen content of plant leaves (Pons and Pearcy, 1994) and the solar energy use efficiency of winter wheat leaf through depression of chlorophyll a/b and non photochemical quenching coefficient (NPQ) (Li et al., 2010). Moreover, the wheat leaf photosynthetic rate was also reduced by depressing the leaf temperature, stomatal conductance and transpiration rate under shading stress (Xu et al., 2013; Dong et al., 2019). Shading stress also resulted in changes to plant carbohydrate accumulation and distribution, causing carbon metabolism imbalance, and eventually led to yield reduction (Grashoff and d'Antuono, 1997; Mu et al., 2010). It has been reported that K can improve crop leaf photosynthesis and enhance the assimilation accumulation and translocation (Verspreet et al., 2013; Wani et al., 2014). Most studies mentioned above were focused on the effect of light intensity or K on the photosynthesis and dry matter production characteristics in wheat, with very little investigation of the interactions of light intensity and K on those parameters. In the present study, a pot experiment was conducted to investigate the effects of K application rate on winter wheat yield, leaf fluorescence parameters and carbohydrate accumulation and translocation with and without shading treatment after anthesis. The results could provide guidance for increasing winter wheat production under low light intensity stress by fertilization management.

## Materials and Methods

### Experimental Design

The pot experiment was carried out at the Wheat-Maize Rotation Nutrition and Fertilizer Application Experimental Station in North China from October 2014 to May 2015. The experimental station is located at 112°42'E−114°14'E, 34°16'N−34°58'N, where it experiences a frost-free period of about 220 days and 2,400 h sunshine each year. Temperature varied from -3°C to 35°C during winter wheat growth period and the average is from 9°C to 11°C. The soil was sandy loam texture with 14% clay, collected from the top layer of the field at the experimental station. The basic physical and chemical properties of the soil were as follows: pH, 7.84; total nitrogen, 960 mg kg^-1^; available nitrogen, 28.4 mg kg^-1^; Olsen-P, 18.2 mg kg^-1^; available K, 66.9 mg kg^-1^; and organic matter, 13.6 g kg^-1^. The soil was air-dried and passed through a 5-mm mesh, and then 10.0 kg of soil was weighed into each polyethylene pot (30 cm tall, 30 cm diameter). K application rate and shading level with 5 × 2 levels in a fully balanced design was employed in this experiment. Five K application rates were included 0 (K0), 50 (K50), 100 (K100), 150 (K150), and 250 (K250) mg K_2_O kg^-1^ soil in the form of KCl, respectively. Two shading levels included no shading (NS), and shading from the 1st day to the 10th days after anthesis (shading at early filling stage for 10 days, SE). The shading net used in the experiment provided a 60% shading rate throughout the experiment. According to data from National Meteorological Information Center (http://data.cma.cn/site/index.html), the average daily minimum temperature in 10 days of shading was 13.7°C, and the maximum temperature was 24.1°C. To fully meet the requirements of wheat for nutrients, 200 mg N kg ^-1^ soil in form of urea and 100 mg P_2_O_5_ kg^-1^ soil in form of Ca(H_2_PO_4_)_2_ were also applied to each pot, respectively. Twelve seeds of wheat cultivar Pingan7 (Zhu99021) were planted per pot on October 7 and thinned to six seedlings after emergence. Plants were sampled at anthesis and maturity for biomass and yield, respectively. To analyze physiological indexes, plants were sampled at 10 and 25 days after anthesis. In this experiment, the valid number of independent biological replication was five for each set of values in statistics, so a total of 175 pots were used in this experiment. During the growth period, the pots were placed outdoors. When it was rain, the rain shelter covered pots. Before wheat anthesis, all the pots were arranged randomly with frequent position changes; at anthesis, these pots were divided into two blocks with shading or not. General management for pest control and irrigation were conducted as necessary. The experiment design is shown in **Supplementary Figure**.

### Plant Biomass and Yield Analysis

The above ground parts of the plants were sampled at anthesis and maturity and were divided into leaf, leaf sheath, stem and seeds. The fresh samples were dried at 105°C for 30 min to inactivate enzymes, then continuously dried at 65°C until the weight remained unchanged. The number of spikes per pots was counted and ten spikes per pot were selected to count the grain number of spikes. After the seed dried, thousand kernel weight and yield were calculated.

### Caculation of Related Indexs

The following formulas were adopted from Cheng et al. (2019) for the calculation of the relevant indicators:

DMTA=(DMA−A)−(DMA−VO−M),

DMTA: dry matter translocation amount from pre-anthesis vegetative organs to grains; DMA-A: total dry matter accumulation at anthesis; DMA-VO-M: dry matter accumulation in vegetative organs at maturity.

DMTP=DMTA×100/DMA−A,

DMTP: the percentage of dry matter translocation amount from pre-anthesis vegetative organs to grains; DMTA: dry matter translocation amount from the pre-anthesis vegetative organs to grains; DMA-A: total dry matter accumulation at anthesis.

### Leaf Spad Value and Chlorophyll Fluorescence Parameters Analysis

At 10 and 25 days after anthesis, five uniform representative main stems were selected in each pot to measure the SPAD value and chlorophyll fluorescence parameters of flag leaves. The SPAD values were measured in the middle of the flag leaves and the average of the five selected flag leaves represented the value of the pot. SPAD-502 (Minolta Camera Co. Ltd., Japan) was used in the experiment.

Chlorophyll (Chl) fluorescence was measured with an FMS-2 type modulated chlorophyll fluorometer (Hansatech, UK). After dark-adaptation of samples for 20 min, the initial fluorescence (F_o_) was measured with weak modulated irradiation (<0.1 µmol m^-2^ s^-1^). A 700-ms saturating flash (>6,000 µmol m^-2^ s^-1^) was applied to determine the maximum Chl fluorescence yield (Fm) and Fv/Fm. Immediately, the leaf was adapted in natural light for 20 min until fluorescence stabilized sufficiently to record Fs. Following this, another saturation flash (>6,000 µmol m^-2^ s^-1^) was applied and then Fm′ was determined. After the flash, the leaves were shaded, far-red irradiation was given after dark adaptation for 3 s, and F_o_′ was determined after 5 s. Other fluorescent parameters were calculated as follows: qP= (F_m_′– F_s_)/(F_m_′– F_o_′); qN= (F_m_– F_m_′)/F_m_. Each indicator was measured three times per flag leaf, and the average value represented the data of the flag leaf. And the average value of the five selected flag leaves represented the value of the pot.

### Fructan and Soluble Total Sugar Contents Analysis

Wheat plants in each treatment were harvested at 10 and 25 days after anthesis, respectively, and the plant samples were divided into stem, leaf sheath and seeds. Dry weights were analyzed. Total soluble sugar contents of each part were determined by anthranone colorimetry. The fructan contents were analyzed according to the method of Verspreet et al. (2013).

### Statistical Analysis

Normality of the population distribution and homogeneity of variances were demonstrated by Shapiro-Wilk test and Levene's Test, respectively. A three-way repeated measures ANOVA was applied to detect the significance of difference in the main effects of K application rate (K0, K50, K100, K150, and K250), shading level (NS, SE) and sampled time (10 and 15 days after anthesis) and in their interaction effects for leaf SPAD, chlorophyll fluorescence parameters, grain soluble sugar content, sheath and stem fructan content. A two-way ANOVA was carried out to analyze the significance of difference in the main effects of K application rate, shading level and in their interaction for the following values: TDM-M (total dry matter accumulation of whole plant at maturity), DMTA, DMTP, grain yield, TKW (thousand kernel weight), GN/S (grain number per spike) and SN/P (spike number per pot). The multiple comparison among the mean values of main effect or among the mean values of the interaction effect were determined with a least significant difference (LSD) test (*P* < 0.05). All of the statistical analyses were performed using SPSS 19.0.

## Results

### Effects of K Application Rate and Shading Treatment on Winter Wheat Yield

Two-way ANOVA revealed significant interactive effects of K × S on wheat grain yield and its components (*P* < 0.01) (**Table 1**).

**Table 1:** *F*-values from two-way ANOVA on the effects of potassium rate (K0, K50, K100, K150, and K250) and shading treatment (no shading, shading 10 days after anthesis) on DMA-VO-M, DMTA, DMTP, grain yield, TKW, GN/S, and SN/P.

Variation	d.f.	DMA-VO-M	DMTA	DMTP	Yield	TKW	GN/S	SN/P
K application rate (K)	4	152.4**	8.6**	6.2**	250.8**	39.6**	22.3**	55.0**
Shading (S)	1	2539.4**	6.0*	17.5**	2107.9**	5365.2**	4505.3**	2.5NS
K × S	4	20.2**	1.3NS	3.5*	250.7**	45.6**	5.6**	28.0**

*, ** represent the test significant at the 5% level (P < 0.05) and 1% level (P < 0.01), respectively; NS, test non-significant at the 5% level. DMA-VO-M, Dry matter accumulation in vegetative organs at maturity; DMTA, Dry matter translocation amount; DMTP, Dry matter translocation percentage; TKW, Thousand kernel weight; GN/S, Grain number per spike; SN/P: Spike number per pot.

**Figure 1:**
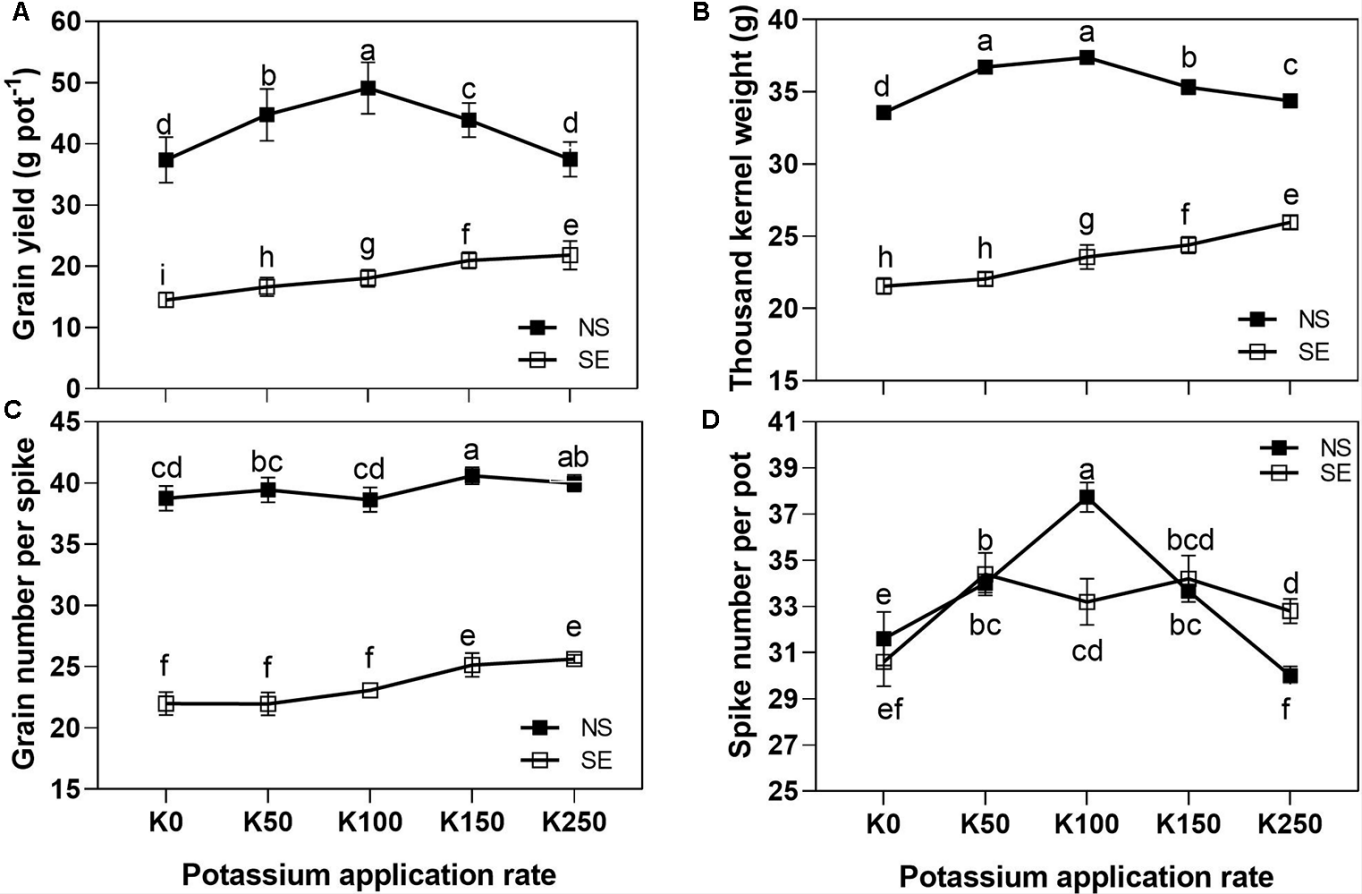
Wheat yield and its components in different treatments. The error bars stand for standard deviations of the means of five independent biological replicates. For each trait, different letters represent significant difference from each other according to two-way ANOVA after testing with least significant difference (LSD) multiple comparison *(P < 0.05)*. NS, no shading treatment; SE: shading from the 1st day to the 10th day after anthesis (shading at early filling stage for 10 days, SE); Five potassium rate: 0 (K0), 50 (K50), 100 (K100), 150 (K150), and 250 (K250) mg K_2_O kg^-1^ soil in the form of KCl.

Under normal light, the wheat yield, TKW and spike number were first increased, and then decreased as K application rate rose. Values reached the maximum at K100 treatment and were significantly higher than in other K application rates, with the exception of TKW in K50 (**Figures 1A, B**, **D**). The effect of K application rate on GN/S was not as significant as the effect on yield, TKW and spike number under normal light condition (**Figure 1C**). Under shading treatment, the wheat yield, TKW and grain number per spike were progressively increased as the K application rate increased, and reached the maximum at K250 treatment. The spike number significantly increased at K50 compared to K0, whereas it did not significantly change as K application rate increased further (**Figure 1D**). At each K application rate, the grain yield, TKW and grain number were sharply decreased in the shading treatment compared with NS condition (**Figure 1**). The results indicated that the application of medium K application rate could effectively improve winter wheat yield under normal light condition. However, under the shading treatment, it was necessary to apply a higher application rate of potassium compared to normal light condition in order to improve winter wheat yield more effectively.

### Effects of K Application Rate and Shading Treatment on SPAD values

The variance analysis showed that the interaction effects of K × S on the SPAD value of flag leaves were significant (*P* < 0.01) (**Table 2**).

**Table 2:** *F*-values from three-way repeated measures ANOVA on the effects of potassium rate (K0, K50, K100, K150, and K250), shading treatment (no shading, shading 10 days after anthesis) and different sampled time (10 and 15 days after anthesis) on leaf SPAD, chlorophyll fluorescence parameters, grain soluble sugar content, sheath and stem fructan content.

Variation	d.f.	SPAD	F_0_	qP	qN	ETR	GSSC	ShFC	StFC
K application rate (K)	4	113.7**	162.5**	187.4**	2740.7**	745.9**	296.4**	179.8**	102.2**
Shading (S)	1	337.4**	1320.2**	167.9**	919.5**	3149.3**	3341.5**	1102.5**	1718.1*
Time (T)	1	1921.7**	9.8**	524.7**	730.5**	652.1**	2186.6**	122.7**	266.9*
K × S	4	42.2**	75.4**	3.2*	221.4**	63.8**	92.5**	58.3**	33.0**
K × T	4	4.3**	10.8**	133.5**	31.3**	154.1**	18.5**	3.7*	65.0**
S × T	1	33.3**	109.8**	0.1NS	256.3**	180.3**	259.1**	33.8**	563.6**
K × S × T	4	1.0NS	34.7**	5.8**	31.3**	18.7**	9.8**	4.2**	22.5**

*, ** represent the test significant at the 5% level (P < 0.05) and 1% level (P < 0.01), respectively; NS, test non-significant at the 5% level. GSSC, Grain soluble sugar content; ShFC, Sheath fructan content; StFC, Stem fructan content.

**Figure 2:**
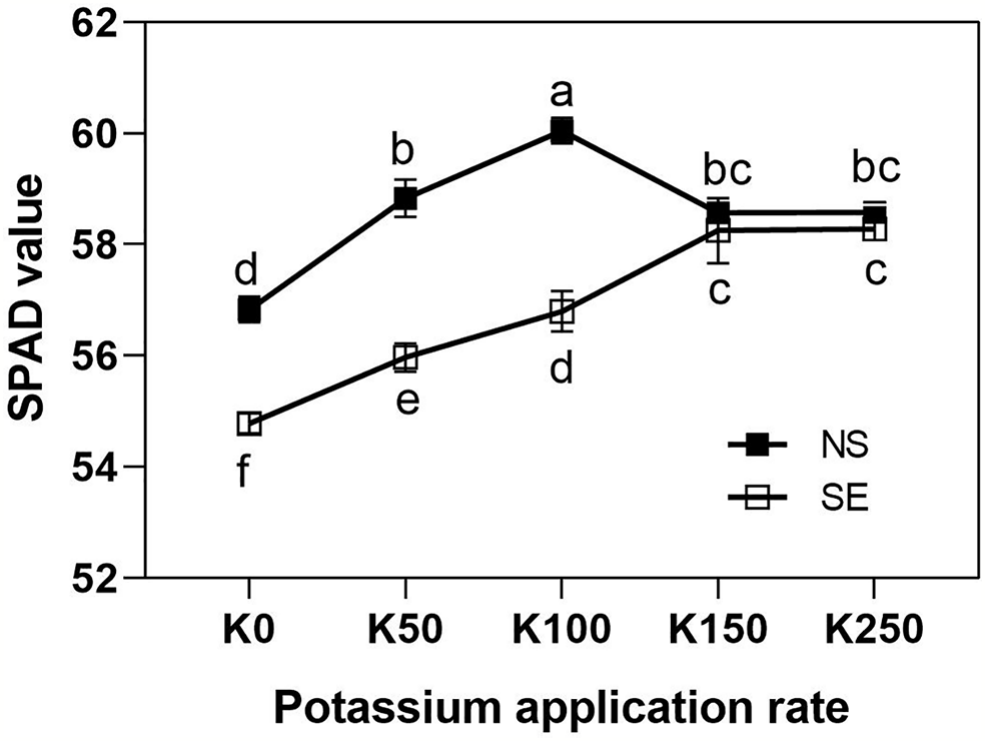
The SPAD values of flag leaves of winter wheat under different treatments. Values are means of SPAD values of 10 and 25 days after anthesis. The error bars stand for standard deviations of the means of five independent biological replicates. Different letters represent significant difference from each other according to two-way ANOVA followed by least significant difference (LSD) multiple comparison (*P* < 0.05). NS: no shading treatment; SE: shading from the 1st day to the 10th day after anthesis (shading at early filling stage for 10 days, SE); Five potassium rate: 0 (K0), 50 (K50), 100 (K100), 150 (K150), and 250 (K250) mg K_2_O kg^-1^ soil in the form of KCl.

The effect of K application rate on SPAD value was different between SE and NS treatment. Under normal light, SPAD values were first increased and then decreased as K application rate rose and peaking at K100 treatment (**Figure 2**). However, under shading treatment, the SPAD values were continuously increased along with the increase of K application rate and peaking at K150 and K250 treatment which was close to NS treatment (**Figure 2**). Shading treatment sharply reduced flag leaves SPAD values under K0, K50, and K100 treatment compared with normal light, whereas there was no significant difference under K150 and K250 condition. It was indicated that because of the interaction effect of K × S, higher K application rate was advantageous to improve leaf chlorophyll content under shading stress condition compared with NS condition.

### Effects of K Application Rate and Shading Treatment on Chlorophyll Fluorescence Parameters

Three-way repeated measures ANOVA revealed significant interactive effects of K × S × T on chlorophyll fluorescence parameters (F0, qP, qN, and ETR) of flag leaves (*P* < 0.01) (**Table 2**).

**Figure 3:**
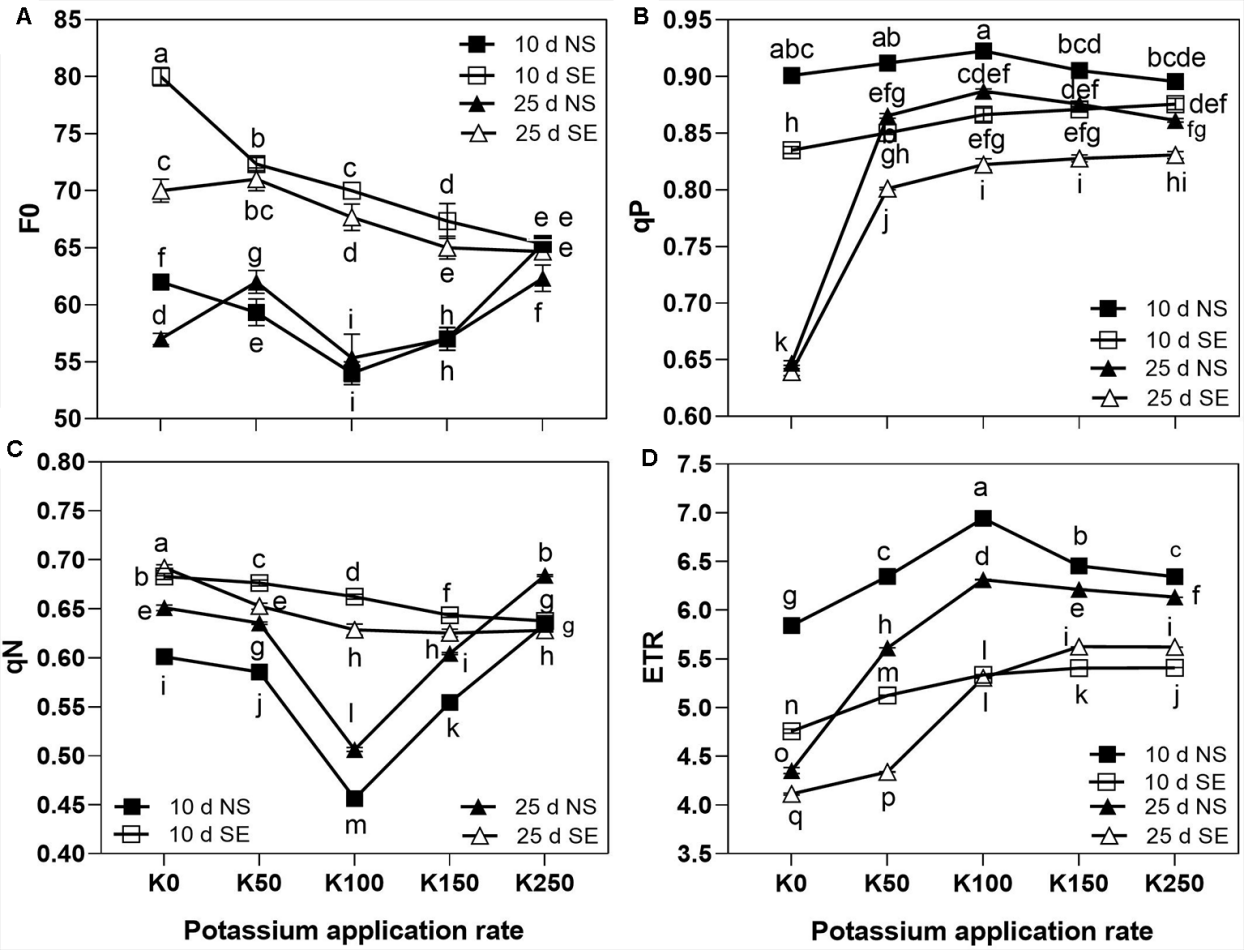
The chlorophyll fluorescence characteristics of winter wheat flag leaves under different treatments. The error bars stand for standard deviations of the means of five independent biological replicates. For each trait, different letters represent significant difference from each other according to three-way repeated measures ANOVA followed by least significant difference (LSD) multiple comparison *(P < 0.05)*. NS: no shading treatment; SE: shading from the 1st day to the 10th day after anthesis (shading at early filling stage for 10 days, SE); Five potassium rate: 0 (K0), 50 (K50), 100 (K100), 150 (K150), and 250 (K250) mg K_2_O kg^-1^ soil in the form of KCl.

The effect of K application rate on F_0_ was different between SE and NS treatment. At 10 days after anthesis, F_0_ was first decreased and then increased as K application rate arose with values reaching the lowest point in K100 treatment under normal light. However, under SE treatment, F_0_ continuously decreased along with the increase of K application rate and at K250 treatment there was no difference between the SE and NS treatment (**Figure 3A**). At 25 days after anthesis, the effect of K application rate on F_0_ was different from that at 10 days under NS condition, which firstly increased from K0 to K50 and then showed a similar change with 10 days. Under SE condition, F_0_ also firstly increased from K0 to K50 and then decreased along with the increase of K application rate. Shading treatment remarkably increased the flag leaf F_0_ compared with NS treatment at all K application rate with the exception of K250 on the both tested days (**Figure 3A**). It indicated that higher K application rate was beneficial to reduce F_0_ under SE condition compared with NS condition due to the interaction effect of K × S × T (**Table 2**).

At the 10 days after anthesis, the values of qP were first increased and reached the highest values at K100, then decreased as the K application rate increased under NS condition. However, under SE condition qP continuously increased along with the increase of K application rate (**Figure 3B**). Shading stress significantly reduced qP values under the same K application rate at 10 days after anthesis. But there was no difference of qP between NS and SE at K0 treatment at 25 days after anthesis. The values of qP at 25 days after anthesis were decreased compared to 10 days under both NS and SE treatment, especially in K0 treatment with the reduction of 23.4% and 27.1%, respectively (**Figure 3B**).

At 10 days after anthesis, the values of qN were first decreased with the minimum value in K100 and then significantly increased as the K application rate rose under NS condition. However, it progressively decreased along with the increase of K application rate under SE treatment (**Figure 3C**). Compared to NS condition, shading stress remarkably increased the values of qN under the same K application rate with the exception of K250 at 10 days after anthesis and K150 at 25 days after anthesis. With the prolongation of time after anthesis, the values of qN were increased under NS condition, and the K effect on it was similar with the 10 days' effect. Under SE condition, the values of qN were decreased along with the increase of K application rate at 25 days after anthesis (**Figure 3C**). It was suggested that winter wheat needs more K to effectively reduce the rise of qN caused by shade stress.

Under NS condition at 10 days after anthesis, as the K application rate increased, the values of ETR were first increased and reached the highest values at K100, then decreased. However, under shading stress, the ETR value constantly increased as K fertilizer application rate increased at 10 days after anthesis. Shading treatment resulted in the remarkably decrease of ETR value under the same K application rate compared with NS condition (**Figure 3D**). At 25 days after anthesis, the values of ETR were decreased compared to 10 days, whereas the effect of K on it was similar to the 10 days' effect under NS condition. Under SE condition, the values of ETR were also constantly increased as K fertilizer application rate increased at 25 days after anthesis. Moreover, the difference between NS and SE was gradually narrowed as the K application rate rose (**Figure 3D**). It was indicated that under shading stress, higher K application rate was conducive to increase ETR compared with NS condition.

### Effects of K Application Rate and Shading Treatment on Grain Soluble Sugar Content in Winter Wheat

As revealed by three-way repeated measures ANOVA, the interaction effects of K × S × T on the grain soluble sugar contents (GSSC) of seeds were significant (*P* < 0.01) (**Table 2**).

**Figure 4:**
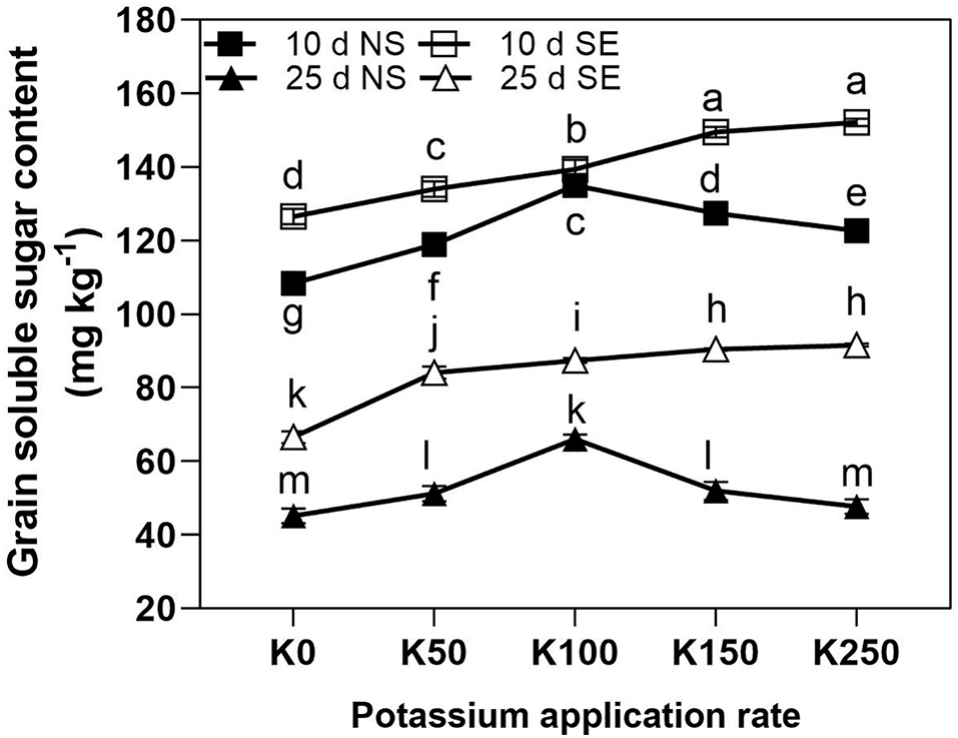
The total soluble sugar content of winter wheat grain under different treatments. The error bars stand for standard deviations of the means of five independent biological replicates. For each trait, different letters represent significant difference from each other according to three-way repeated measures ANOVA followed by least significant difference (LSD) multiple comparison (*P* < 0.05). NS: no shading treatment; SE: shading from the 1st day to the 10th day after anthesis (shading at early filling stage for 10 days, SE); Five potassium rate: 0 (K0), 50 (K50), 100 (K100), 150 (K150), and 250 (K250) mg K_2_O kg^-1^ soil in the form of KCl.

Under NS condition, the effect of K application on GSSC was similar at 10 days and 25 days after anthesis. They both increased at first and then decreased with the highest value in K100 treatment. Unlike NS condition, the GSSSC of SE treatment showed a continual increase along with the increase of K application rate (**Figure 4**). Shading stress significantly increased the GSSC compared with NS treatment under the same K application rate with the exception of K100 at 10 days after anthesis. The GSSC was sharply reduced at 25 days after anthesis compared to at 10 days in the same K application rate under NS and SE conditions (**Figure 4**).

### Effects of K Application Rate and Shading Treatment on Fructan Contents in Sheath and Stem in Winter Wheat

Three-way repeated measures ANOVA revealed significant interactive effects of K × S × T on the fructan content in sheath (ShFC) and stem (StFC) (*P* < 0.01) (**Table 2**).

**Figure 5:**
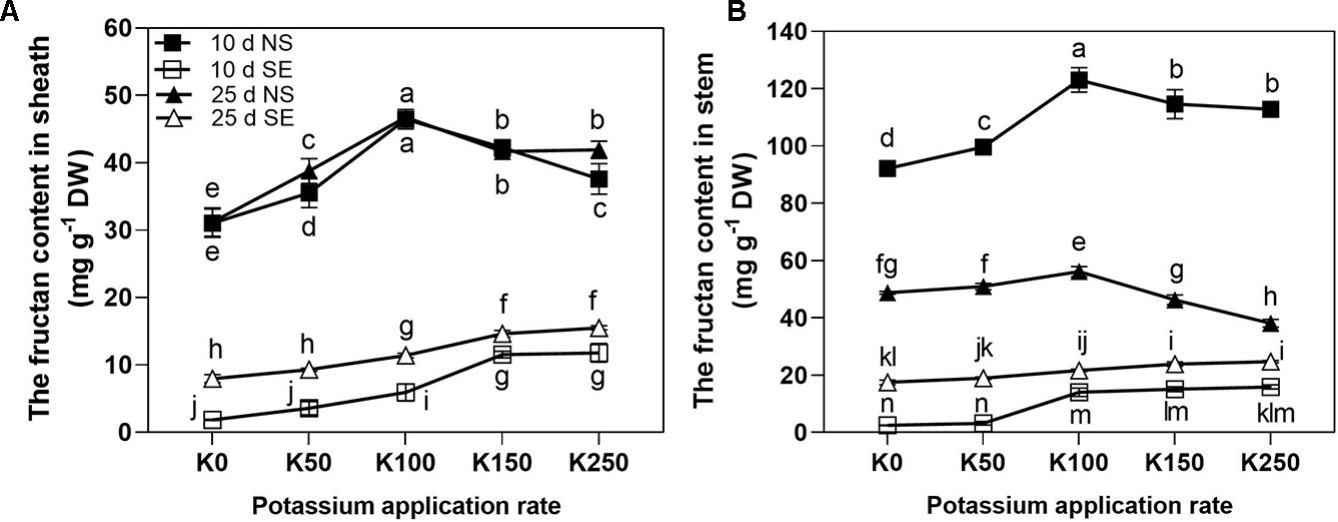
The fructan contents in sheath **(A)** and stem **(B)** under different treatments. The error bars stand for standard deviations of the means of five independent biological replicates. For each trait, different letters represent significant difference from each other according to three-way repeated measures ANOVA followed by least significant difference (LSD) multiple comparison (*P* < 0.05). NS: no shading treatment; SE: shading from the 1st day to the 10th day after anthesis (shading at early filling stage for 10 days, SE); Five potassium rate: 0 (K0), 50 (K50), 100 (K100), 150 (K150), and 250 (K250) mg K_2_O kg^-1^ soil in the form of KCl.

Shading stress sharply reduced the fructan content both in the sheath and stem compared with NS conditions under all the K treatments (**Figure 5**). At 10 days after anthesis, ShFC was increased first peaking at K100 rate and then decreased as the K application rate rose under NS condition, whereas it was showed a continual increase along with the increase of K application rate under SE condition (**Figure 5A**). At 25 days after anthesis, the effect of K application rate on ShFC under NS condition was also firstly increased, then decreased, but values stabilized at K150 and K250. Under SE condition, ShFC of 25 days was also continually increased along with the increase of K application rate. There was no conspicuous difference of ShFC between 10 and 25 days in the same K application rate under NS condition with the exception of K50 and K250. However, under SE condition, ShFC of 25 days was higher than that of 10 days (**Figure 5A**). At 10 days after anthesis, the effect of K application rate on StFC similar with ShFC both under NS and SE condition (**Figure 5B**). The StFC of NS treatment was decreased significantly at 25 days after anthesis compared with 10 days (**Figure 5B**). At 25 days after anthesis, the effect of K application rate on StFC under NS condition was firstly increased peaking at K100, and then significantly decreased. However, under SE condition, the StFC increased at K100 and then kept stable even the K application rate was increased (**Figure 5B**).

### Effects of K Application Rate and Shading Treatment on Winter Wheat Dry Matter Accumulation and Translocation After Anthesis

Two-way ANOVA revealed significant interactive effects of K × S on DMA-VO-M and DMTP (*P* < 0.05 or 0.01). Only the main effect of K or shading treatment (S) on DMTA was significant (*P* < 0.05 or 0.01) (**Table 1**).

Plant biomass of winter wheat at anthesis stage (DAM-A) was increased with K, peaking at K100 rate, and then decreased with the increase of K application rate (**Table 3**). The response of DAM-VO-M to changes in K rate showed a similar trend to DAM-A both in NS and SE treatment (**Table 3**). Shading treatment significantly reduced the DAM-VO-M under the same K application rate compared with the NS condition. Under NS condition, the trend of DMTA with the increase of K application rate was similar to that of DMA-A and DAM-VO-M. Shading stress enhanced the DMTA compared with NS treatment under K0 and K50, while there was no significant difference between other K application rates compared with NS. K application significantly increased the DMTP, while there was no significant difference between K100, K150 and K250 in NS treatment (**Table 3**). Shading stress significantly enhanced DMTP, and the higher the K application rate, the more significant the effect (**Table 3**). It was indicated that shading stress reduced the dry matter accumulation, whereas the dry matter translocation rate from vegetative organs to grains was significantly increased compared to NS. Moreover, because of the interaction effect of K × S, the increased effect of DMTP under SE was enhanced as K application rate increased (**Table 1** and **Table 3**).

**Table 3:** Effect of potassium rate and shading-stress on plant dry matter accumulation and translocation in wheat.

K application rate	DMA-A (g pot^-1^)	DMA-VO-M (g pot^-1^)		DMTA (g pot^-1^)		DMTP (%)	
	NS	NS	SE	NS	SE	NS	SE
K0	74.7 ± 7.0d	53.3 ± 3.2c	44.7 ± 1.3f	21.4 ± 1.8e	30.0 ± 3.5bc	28.7 ± 1.7d	40.2 ± 2.9abc
K50	79.0 ± 4.8c	53.5 ± 1.6c	46.3 ± 2.3ef	25.5 ± 5.1d	32.7 ± 5.1bc	32.0 ± 2.0cd	41.2 ± 4.1bc
K100	100.6 ± 7.4a	59.7 ± 6.1b	62.3 ± 1.5a	41.0 ± 7.3a	37.8 ± 6.5ab	40.5 ± 2.8abc	38.3 ± 1.0bc
K150	87.1 ± 3.5b	53.1 ± 3.7c	48.3 ± 2.5de	34.0 ± 5.3abc	38.8 ± 2.7ab	38.9 ± 1.4bc	44.5 ± 1.3ab
K250	84.2 ± 5.6bc	49.4 ± 1.4d	45.2 ± 3.6f	34.8 ± 3.1abc	39.0 ± 1.7ab	41.3 ± 2.8bc	46.3 ± 1.2a

Values are means ± SD of five independent biological replicates.For each trait, different letters represent significant difference from each other according to two-way ANOVA after testing with least significant difference (LSD) multiple comparison (P < 0.05). NS, no shading treatment; SE, shading from the 1st day to the 10th days after anthesis (shading at early filling stage for 10 days, SE); Five potassium rate, 0 (K0), 50 (K50), 100 (K100), 150 (K150) and 250 (K250) mg K_2_O kg_-1_ soil in the form of KCl; DMA-A, Dry matter accumulation at anthesis (before shading treatment); DMA-VO-M, Dry matter accumulation in vegetative organs at maturity; DMTA, Dry matter translocation amount; DMTP, Dry matter translocation percentage.

## Discussion

Plants often have to cope with altered light conditions, which in leaves induced various physiological responses from photosynthetic acclimation to leaf senescence. Under low light, the activity of the carbon-concentrating mechanism generally decreases, associated with an increase in leakiness, the ratio of CO_2_ retro diffusing from the bundle sheath relative to C_4_ carboxylation (Bellasio and Griffiths, 2014). The magnitude of the yield reduction depends on the phase of plant development (Che et al., 2005; Cui et al., 2015), and the greatest reductions occur when shading is imposed during the early reproductive phase (González et al., 2011; Xu et al., 2016). There was a critical impact on barley nitrogen uptake amount, grain yield and grain protein yield from shading at the early stage of grain filling (Grashoff and d'Antuono, 1997). The present research also demonstrated that shading during 0–10 days after anthesis significantly reduced the wheat grain yield (**Figure 1A**).

Grain number per spike and TKW are two key factors which reflect wheat yield, and the nutrient supply for panicle during anthesis demonstrated an important link with the formation of spike grain number (Ferrante et al., 2010). Previous studies showed that the major reason for shading decreasing grain yield as reflected in the reduced TKW was due to depressed plant nitrogen accumulation, leaf photosynthetic rate and grain filling rate (Jia et al., 2011; Xu et al., 2013; Li et al., 2017). The reduction of grain weight had most serious impact on wheat yield (Mu et al., 2010), which was dependent on the number and size of endosperm cells. Generally, the maximum endosperm cell proliferation rate period was during the 5 to 10 days after anthesis, and the endosperm cell size was associated with the soluble sugar content (Nadaud et al., 2010). So shading stress at early grain filling stage could depress endosperm cell differentiation, which reduced its storage capacity. This might have resulted in yield decrease for the shading stressed winter wheat in the present study. On the other hand, the conversion of soluble sugar to starch was blocked, which led to the reduction of grain size and ultimately grain yield under this stressed condition. In this study, the blockage to soluble sugar conversion to starch was reflected in significantly increased total soluble sugar content in the grain of winter wheat after shading for 10 days after anthesis (**Figure 4**).

Reduction in light intensity and changes in light spectrum under shading have been shown to alter chloroplast ultrastructure and chlorophyll contents (Kasemir, 1979; Mauro et al., 2011; Deng et al., 2012). In our study, shading treatment resulted in a decrease in SPAD value (**Figure 2**). Chlorophyll fluorescence parameters were often used to evaluate the function of photosynthetic apparatus and carbon assimilation rate due to its sensitivity, convenience and nonintrusive characteristics (Huang et al., 2004; Deng et al., 2012; Cui et al., 2015). Our results showed that chlorophyll fluorescence parameters of F_0_ increased significantly after shading stress for 10 days at anthesis stage. As an effective indicator of the proportion of open PSII reaction centers, the qP describes the quantum use for the photochemical process of a healthy leaf and high qP is helpful to transport electrons in the center. In the present study, shading stress significantly reduced qP value (**Figure 3B**). It indicated that a long-term sustained severely low irradiance was harmful to the growth of wheat. The qN represents the energy which cannot be utilized to transport photosynthetic electrons being dissipated harmlessly as heat energy from PSII antennae (Müller et al., 2001). It have been reported that the higher qN in slight (e.g. 50%) shade treated plants showed that the energy absorbed in the physiological range of irradiance was much higher than photochemical utilization, which may cause inhibition of photosynthetic capacity (**Figure 3C**). The enhanced qN value demonstrated again that the winter wheat plants were not suitable for a long-term sustained shading environment.

It has been demonstrated that shading before flowering stage had larger effects on the plant K absorption than on the N and P absorption (Cui et al., 2013; Frantz, 2013), which indicated that plant will be suffered in K deficiency under shading stress. As a result, high K application rate could improve wheat K nutrition status under shading stress which was beneficial for photosynthesis. The present results showed that there was interaction of K × S on most of all investigated traits and higher K application rate could relieve the decrease of wheat yield under shading stress. It has been demonstrated that K plays a central role in maintenance of photosynthesis and related processes (Terry and Ulrich, 1973; Collins and Duke, 1981; Jin et al., 2011). Zhao et al. (2001) demonstrated that net photosynthetic rate (P_N_) of the uppermost fully expanded main-stem leaves of K-deficient cotton was only 23% of the control plants receiving a full K supply. The present study showed that chlorophyll SPAD value, chlorophyll parameters of qP and ETR in wheat flag leaves were significantly improved, whereas chlorophyll fluorescence parameters F_0_ and qN were reduced along with K application rate increasing after shading stress (**Figures 2** and **3**). It indicated that because of the interaction of K × S, high K application rate can reduce the damage of shading stress on winter wheat through alteration of chlorophyll fluorescence parameters to enhance photosynthesis.

K can effectively coordinate the relationship between source and sink of starch synthesis, including the synthesis, transport and transformation of photosynthate (Li et al., 2012). Stem and leaf sheath of wheat were the main storage organs for photosynthetic products which exist mainly in the form of water soluble carbohydrates (Verspreet et al., 2013). It has been reported that the contribution of carbohydrates stored in stem to grain yield was greater than that stored in the sheath (Schnyder, 1993). Moreover, fructan played the most important role in yield among different types of carbohydrates, and its accumulation and translocation were the main source of grain yield (Schnyder, 1993; Joudi et al., 2012; Meguro-Maoka and Yoshida, 2016). Under stress conditions, water soluble carbohydrates had important significance in maintaining high grain-filling rate to keep high yield of wheat (Schnyder, 1993). In the present study, we observed that shading stress significantly reduced the fructan in wheat stem and sheath. However, fructan content was continuously increased along with the increase of K application rate under shading stress (**Figure 5**). This indicated that because of the interaction of K × S, application of more K fertilizer (K150, K250) could relieve the reduction of water soluble carbohydrates of stem and sheath owing to the shading stress, which was advantageous for maintaining higher grain filling rate and achieving higher TKW and yield. In addition, the fructan content in winter wheat had significant correlation with cold resistance and water logging resistance (Takagi et al., 2016). Consequently, the increase of fructan content in wheat stem and sheath contributed by higher K application rate under shading stress might enhance the resistance to other stresses.

## Conclusion

The present work demonstrated that there was an interaction effect of K × S on photosynthesis and photosynthate translocation of wheat. As the result of this interaction, compared to NS condition, higher K application rate (150 and 250 mg K_2_O kg^-1^ soil) was advantageous to effectively alleviate the damage on the winter wheat photosynthetic system caused by shading stress, increase DMTP, leading to the improvement of the grain yield of shading stressed winter wheat.

## Data Availability Statement

The raw data supporting the conclusions of this manuscript will be made available by the authors, without undue reservation, to any qualified researcher.

All datasets [generated/analyzed] for this study are included in the manuscript and the supplementary files.

## Author Contributions

YanH and JT conceived the original research. YW performed the most experiments. ZZ prepared with the plant materials and test soil. YL and YuH assisted with the management of pot culture and physiological analysis. YW and YanH wrote the article.

## Funding

This work was supported financially by National Key R&D Program of China (Project Nos. 2018YFD0300706 and 2018YFD0200600) and Henan Agricultural University Science and Technology Innovation Fund (KJCX2015A16).

## Conflict of Interest

The authors declare that the research was conducted in the absence of any commercial or financial relationships that could be construed as a potential conflict of interest.

## Supplementary Material

Supplementary Figure Experiment design diagram. The sown time, anthesis time, sampled time and harvested time of wheat are shown in the figure.
