# Supplementary figures and images for: High Potassium Application Rate Increased Grain Yield of Shading-Stressed Winter Wheat by Improving Photosynthesis and Photosynthate Translocation
Source: Front Plant Sci. 2020 Feb 28;11:134. doi: 10.3389/fpls.2020.00134 (PMC7058633; doi:10.3389/fpls.2020.00134)

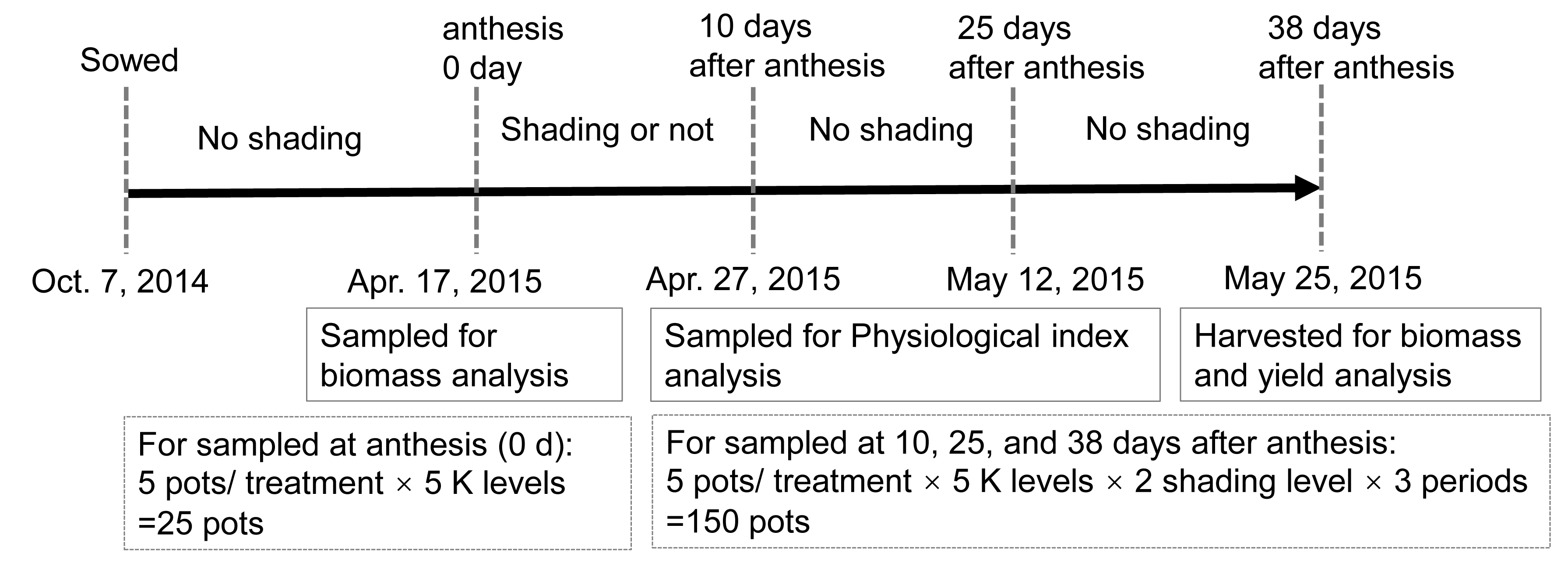

Supplement: Supplementary Figure — Experiment design diagram. The sown time, anthesis time, sampled time and harvested time of wheat are shown in the figure. [file Image_1.tif]
